# Ultimate Drivers and Proximate Correlates of Polyandry in Predatory Mites

**DOI:** 10.1371/journal.pone.0154355

**Published:** 2016-04-21

**Authors:** Peter Schausberger, J. David Patiño-Ruiz, Masahiro Osakabe, Yasumasa Murata, Naoya Sugimoto, Ryuji Uesugi, Andreas Walzer

**Affiliations:** 1 Group of Arthropod Ecology and Behavior, Department of Crop Sciences, University of Natural Resources and Life Sciences, Vienna, Austria; 2 Department of Behavioural Biology, University of Vienna, Vienna, Austria; 3 Laboratory of Ecological Information, Graduate School of Agriculture, Kyoto University, Oiwake-cho, Kitashirakawa, Sakyo-ku, Kyoto, Japan; University of Thessaly, GREECE

## Abstract

Polyandry is more widespread than anticipated from Bateman’s principle but its ultimate (evolutionary) causes and proximate (mechanistic) correlates are more difficult to pinpoint than those of polygyny. Here, we combined mating experiments, quantification of reproductive traits and microsatellite genotyping to determine the fitness implications of polyandry in two predatory mite species, where males are highly polygynous (up to 45 fertilized females during life), whereas females range from monandry to various polyandry levels. The medium-level polyandrous (up to eight male mates possible) *Neoseiulus californicus* received clear direct and indirect benefits: multiply mated females produced more offspring with higher survival chances over longer times than singly mated females. In contrast, singly and multiply mated females of the low-level polyandrous (commonly two male mates at maximum) *Phytoseiulus persimilis* produced similar numbers of offspring having similar survival chances. In both species, multiple mating resulted in mixed offspring paternities, opening the chance for indirect fitness benefits such as enhanced genetic compatibility, complementarity and/or variability. However, the female re-mating likelihood and the paternity chance of non-first male mates were lower in *P*. *persimilis* than in *N*. *californicus*. Regarding proximate factors, in both species first mating duration and female re-mating likelihood were negatively correlated. Based on occasional fertilization failure of first male mates in *P*. *persimilis*, and mixed offspring paternities in both species, we argue that fertilization assurance and the chance to gain indirect fitness benefits are the ultimate drivers of polyandry in *P*. *persimilis*, whereas those of *N*. *californicus* are higher offspring numbers coupled with enhanced offspring viability and possibly other indirect fitness benefits. Overall, the adaptive significance and proximate events well reflected the polyandry levels. Our study provides a key example for linking behavioral experiments, quantification of reproductive traits and paternity analysis via offspring genotyping to explain the evolution of differing levels of polyandry.

## Introduction

Bateman’s principle [1] states that the reproductive success of males is much more variable than that of females. Due to different investment in gamete production [2], males gain in reproductive fitness primarily by increasing the number of females they fertilize, whereas females gain primarily by investment in offspring quality but not number of mates. Thus, females are not expected to mate as frequently as do males. Additionally, the mating act seems to entail higher costs, in terms of loss of energy and time and the risks of physical harm and/or pathogen infection [3], for females than males, selecting females to more carefully trade-off the benefits against the costs of re-mating. However, while Bateman’s principle still holds largely true for intersexual comparisons of mating frequencies, it is now generally acknowledged that polyandry is much more widespread across animal taxa, both vertebrates [4, 5], especially birds, and invertebrates [3, 6, 7], especially insects, than previously anticipated and is no longer considered the exception from the rule. This is also because research revealed that polyandry may not only increase offspring number but may yield diverse other direct (material and non-genetic) and indirect (genetic) benefits [3, 8–11]. Direct material and non-genetic benefits may arise from assuring sufficient sperm supply (more or fresher sperm) to fertilize all available eggs and/or providing adequate (more, sufficient, better) resources (for example through accessory substances or nutrients or habitat features), enhancing or stimulating egg production or individual offspring quality [3]. Indirect genetic benefits result from sperm competition and/or cryptic female choice [12, 13] and are those where genotypic offspring features, due to mixed paternity, enhance the mean offspring fitness, as compared to offspring sired by a single male [9, 10]. Genetic benefits may increase viability [14, 15], compatibility, sexual attractiveness or genetic complementarity and variability of offspring [9, 10]. Some genetic benefits, such as genetic complementarity or variability mitigating inbreeding depression under sib mating, may only become apparent in the 2^nd^ filial generation, i.e. in grand-offspring [9].

Identifying, separating and quantifying direct non-genetic and indirect genetic fitness effects of polyandry (i.e. its ultimate drivers) and scrutinizing associated proximate factors requires combining behavioral, reproductive and molecular analyses. Ultimate refers to the evolutionary causes of a given behavior while proximate refers to the underlying mechanistic factors. Such an approach is particularly important if mating frequencies are flexible and vary within and between species [16], i.e. where some individuals re-mate whereas others mate only once. However, studies investigating behavioral, reproductive and genetic aspects of polyandry in one and the same individuals, allowing linking ultimate drivers and proximate correlates of polyandry, are rare. Such studies are hardly accomplishable in natural settings, but also experimentally challenging in the laboratory, because assessing lifetime reproductive success requires following females until naturally ceasing oviposition. Moreover, clear distinction between direct (immediate) material/non-genetic and indirect genetic benefits can be experimentally difficult because some direct non-genetic effects, such as improved offspring quality, may phenotypically resemble indirect genetic effects. Thus, rigorous examination of polyandry benefits, allowing accounting for indirect genetic mechanisms, must involve genetic paternity analyses. Offspring number and quality may alone be influenced, regardless of fertilization, by the mating process itself or the presence of sperm or substances accompanying sperm. This may, for example, happen through nutrients in the spermatophores/seminal fluids or epigenetic and other non-genetic effects [17, 18] induced by the mating act itself or chemical compounds in the seminal fluid of other mating partners [19], triggering enhancement of egg production or changing maternal behavior affecting foraging and, by that way, better provisioning offspring. Multiple mating by females not changing offspring paternity as compared to singly mating (monandrous) females (i.e. offspring only sired by one male) has recently been termed pseudo-polyandry and only polyandry leading to offspring sired by multiple males is termed true polyandry [20]. This distinction is important because pseudo-polyandry can only exert direct material or non-genetic fitness effects, whereas true polyandry additionally includes indirect genetic effects.

Here, we combined behavioral, reproductive and molecular analyses to determine and characterize the proximate and ultimate factors of polyandry in two sympatrically occurring predatory mite species, *Phytoseiulus persimilis* and *Neoseiulus californicus*. These plant-inhabiting predators constitute natural guilds sharing herbivorous spider mites as prey [21]. Both predators reproduce sexually, are sexually dimorphic with large females and small males, and are pseudo-arrhenotokous, i.e. only daughters are diploid and carry the paternal chromosome set [22]. The main patterns and processes of their mating behaviors are similar, with males actively searching for females but after mate encounter, predominantly females having control whether copulation takes place or not [21]. Both are highly polygynous with a single male fertilizing up to 45 females and thereby siring up to 1500 eggs [23]. Females of both predatory mite species may mate multiply but while *P*. *persimilis* females re-mate only occasionally, with no more than two male mating partners [24, 25], *N*. *californicus* females re-mate commonly, with up to eight male mating partners [21, 26]. Accordingly, we hypothesized that polyandry yields higher fitness benefits in *N*. *californicus* than *P*. *persimilis*, which is proximately reflected in female mating patterns, especially re-mating propensities, and offspring paternities.

## Materials and Methods

We conducted mating experiments, quantified reproductive traits and analyzed offspring paternities using isofemale lines of *N*. *californicus* and *P*. *persimilis*. For each species, the isofemale lines derived from the same source population but, within species, differed in the alleles on several microsatellite loci. To this end, first, we determined polymorphic microsatellite loci in *N*. *californicus* and *P*. *persimilis*, second, established isofemale lines differing in microsatellite alleles, third, conducted the mating experiments and quantified the female lifetime reproductive success (i.e. the total number of viable offspring produced during life), and, fourth, genotyped the offspring of multiply mated females for paternity determination. Isofemale lines used in the mating experiments and for offspring genotyping were equipped with unique sets of alleles allowing clear discrimination from other lines of the same species.

### Determination of polymorphic microsatellite loci

Species-specific polymorphic microsatellite loci were determined using a *P*. *persimilis* population founded with specimens provided by Tomono Agrica Co. Ltd., Shizuoka, Japan, and a *N*. *californicus* population founded with specimens collected from Japanese pear trees in Matsukawa, Nagano Prefecture, Japan. Genomic DNA from 50 adult females each of *P*. *persimilis* and *N*. *californicus* was extracted using the GenomicPrep Cells and Tissue DNA Isolation Kit (GE Healthcare UK, Little Chalfont, Buckinghamshire, UK). DNA was digested with a restriction endonuclease, *Sau* 3A I (Takara Bio Inc., Otsu, Shiga, Japan), ligated into *Sau* 3A I cassettes, and amplified using a cassette primer (Takara Bio Inc.) by polymerase chain reaction (PCR). Amplified fragments ranging from 300 to 1000 bp lengths were hybridized to 5' biotin-labeled oligonucleotide probes (biotin-(CA)_15_) and isolated by means of Streptavidin MagneSphere Paramagnetic Particles (Promega, Madison, WI, USA). Resulting microsatellite-enriched fragments were ligated into a plasmid, pUC118/*Bam* H I, and introduced into *Escherichia coli* DH5α-competent cells (Takara Bio Inc.). Plasmids were sequenced in the 48 transformant *E*. *coli* clones for each species using the BigDye Terminator Cycle Sequencing Kit version 3.1 and the ABI PRISM 3130 Genetic Analyzer (Applied Biosystems, Foster City, CA, USA).

Ten primer sets (microsatellite loci), which are used for the detection of polymorphic loci by fragment analyses, were selected for each species using GENETYX ver. 4.0.2 software (Genetyx Co., Tokyo, Japan).

### Establishing isofemale lines

#### Species origin and rearing

Specimens of *P*. *persimilis* and *N*. *californicus* used to establish laboratory-reared populations originated from Sicily [27]. The predators were reared in piles of *T*. *urticae*-infested bean leaves placed on separate arenas consisting of square plastic tiles (15 x 15 cm) resting on water-saturated foam cubes in plastic boxes half-filled with water. New spider mite-infested leaves were added in 2 to 3 day intervals. The spider mites used as prey for the predators were reared on whole common bean plants, *Phaseolus vulgaris*, at room temperature (20 to 25°C).

#### Generating isofemale lines

To generate isofemale lines of *P*. *persimilis* and *N*. *californicus*, 10 to 15 female deutonymphs were withdrawn from the stock population and singly isolated on detached leaf arenas infested with spider mites. Each leaf arena (~5 x 5 cm) consisted of a detached bean leaf placed upside down on a water-saturated foam cube in a plastic box half-filled with water. Water-saturated tissue strips were used to confine the arena and prevent the mites from escaping. After reaching adulthood, each female was paired with a single mating partner of random age. The offspring of each couple were inbred for three to five generations, lasting about five to eight weeks, and used to establish isofemale lines. Lines with unique sets of alleles, allowing clear discrimination from other lines of the same species, were kept for the experiment. To determine the set of alleles on three polymorphic microsatellite loci, 10 to 15 females of each line were genotyped and only those lines were kept for the experiment where every genotyped female had the same unique set of alleles.

### Mating experiments and quantification of reproductive traits

Five to ten females from each of three isofemale lines of *P*. *persimilis* and *N*. *californicus*, respectively, were placed on separate detached leaf arenas (one arena per isofemale line) and provided with ample spider mite prey to generate eggs giving rise to females used in the experiment. After 2 days, the females were removed and only their eggs were left on the arenas for development. After another 3 to 4 days, the predatory mites had developed to deutonymphs. Female deutonymphs were removed and singly isolated on separate leaf arenas infested by spider mites until molting to adult females. These females constituted the experimental individuals. After reaching adulthood (day 0), each female was offered a single male of random age, randomly withdrawn from a random isofemale line, during three time periods: days 0 to 2, days 9 to 13 and days 17 to 23. During each time period, the mating latency, i.e. the time elapsed until mating occurred, and mating duration were recorded. Males were removed after the copulation had finished or, if no mating occurred, after 3 h. Multiple mates of a given female derived from different isofemale lines. The timing of, and intervals between, male mate offerings were determined in pilot experiments, covering the needs of both species [25, 26] and allowing both the chance to re-mate immediately after the first mating and the chance to re-mate at a later time. The maximum number of successful matings by a female was set to 3 in our experiments. In *N*. *californicus* females, more than two male mates do not result in further increase of the number of eggs laid [26]. The 1^st^ male was offered on day 0. The 2^nd^ male was offered on day 1. If no mating occurred on day 1, another 2^nd^ male was offered on day 2. On days 9 to 13, a 3^rd^ male was offered to the female. If no mating occurred, another 3^rd^ male was offered the next day. On days 17 to 23, only for those female that had not yet mated three times, another 3^rd^ male was offered. If no mating occurred, another 3^rd^ male was offered the next day.

Every other day, beginning with the day following the first copulation of the female, survival of the female and the number of eggs laid were recorded. Eggs were removed and placed on separate arenas infested with spider mites. To minimize cannibalism, the eggs of a given female were placed on several arenas, with grouping eggs from three collections, i.e. laid over a time span of 6 days, on the same arena. Offspring were reared to adulthood to determine their sex. All rearing and (pre)-experimental units were stored in environmental chambers at 25 ± 1°C, 60 ± 5% relative humidity and 16:8 h light:dark. Adult daughters were stored in 95% alcohol in Eppendorf tubes and later subjected to paternity analysis. Due to pseudo-arrhenotoky only daughters carry the paternal chromosomes [22] and can thus be used for paternity analyses. Only females that could be observed until their oviposition ceased naturally (i.e. no eggs laid for three consecutive days) were included in statistical analyses (N = 30 in *P*. *persimilis* and N = 23 in *N*. *californicus*). Female offspring of 11 and 10 multiply mated females of *P*. *persimilis* and *N*. *californicus*, respectively, could be successfully genotyped and subjected to paternity analyses.

### Genotyping by fragment analyses of microsatellite loci

Adult females of the isofemale lines of *P*. *persimilis* and *N*. *californicus* were randomly withdrawn from the rearings and stored in 95% ethanol. The mites were dried on filter paper for 15 min at ~25°C, individually homogenized in 20 μl of lysis buffer (10 mM Tris-HCl, pH 8.0, 100 mM EDTA, 0.5% Igepal CA-630 (Sigma-Aldrich Co., St. Louis, MO, USA), 10 mM NaCl, and 1 mg ml^−1^ proteinase K), and incubated at 65°C for 15 min and at 95°C for 10 min. The homogenate was then diluted with 180 μl of 0.1×TE buffer (1 mM Tris-HCl (pH 8.0), 0.1 mM EDTA) and stored at −25°C until use in PCR as a template DNA solution for fragment analysis.

PCR amplifications were carried out using a TaKaRa PCR Thermal Cycler TP600 (Takara Bio Inc.) as follows: 3 min at 96°C, 40 cycles of 1 min at 95°C, 1 min at 64°C, and 2 min at 72°C, followed by 10 min at 72°C. The 20 μl reaction volume contained 1 μM of fluorescently labeled forward and non-labeled reverse primers, 0.2 mM dNTPs, 10 mM Tris–HCl (pH 8.3), 50 mM KCl, 2.5 mM MgCl_2_, 1 U of TaKaRa Ex Taq (Takara Bio Inc.), and 1 μl of the template DNA solution. PCR products (1 μl) were used for fragment analysis by an ABI 3130–200 genetic analyzer with GeneMapper version 3.5 (Applied Biosystems). Of the ten determined microsatellite loci, three and six loci were polymorphic in the isofemale lines of *N*. *californicus* and *P*. *persimilis*, respectively, used in the mating and paternity experiment. For paternity analyses, in each species two diagnostic loci based on the genotypes of parental mites were used (Table 1). Prior to paternity analyses, we confirmed adherence to the Hardy-Weinberg equilibrium and absence of null allele estimation, respectively, using Cervus 3.0 [28].

**Table 1:** Primer sequences and characteristics of four polymorphic microsatellite loci in the predatory mites *P*. *persimilis* (PP003 and PP005) and *N*. *californicus* (NC019 and NC030).

Locus designation (accession no.)^a^	Primer sequence (5'-3')	Fluorescent label	Melting temperature (°C)	Repeat motif	Sequence size (bp)^b^	Diagnostic alleles used in paternity analyses (bp)^c^
PP003	F: ATGTACGAACCGCGGGAACT	PET	60.8	(CT)_10_	126	122, 124
(LC017803)	R: TTTATGGAGACAGGGACCTGGATG	—	60.9			
PP005	F: GTTGCGAAGGTTGAACCGAA	NED	58.0	(GA)_10_	274	266, 272
(LC017804)	R: CATTTTGCAACGGGCGAGAA	—	58.7			
NC019	F: GGAGTAAAAGGCCACAGTAGGAG	VIC	59.3	(GA)_8_	172	162, 164, 178, 182
(LC017805)	R: CAGCAGGGCCAATAAGTTGGA	—	59.7			
NC030	F: CGACTCACTTTCGAAGCGAGAA	6-FAM	59.5	(AG)_3_GG(AG)_7_	237	229, 235, 237
(LC017806)	R: CTTCAACCGTGGGAACCCAA	—	59.4			

^a^DDBJ (http://www.ddbj.nig.ac.jp/).

^b^refers to populations used for isolation of microsatellite loci.

^c^refers to populations used in mating experiments.

### Statistical analyses

#### Mating and reproductive traits

Statistical analyses were carried out using SPSS 21 (IBM Corp., USA). We used separate generalized linear models (GLM) to compare (1) the mating frequency (number of matings) of females of *N*. *californicus* and *P*. *persimilis* (Poisson distribution, log link; species additionally nested in experimental series), (2) the first and second mating dates, i.e. days after reaching adulthood (Poisson distribution, log link), mating latencies (normal distribution, identity link), and mating durations (normal distribution, identity link) of singly and multiply mated females of both species, (3) the first mating duration (normal distribution, identity link) between females of *P*. *persimilis* and *N*. *californicus* staying singly mated and those re-mating later, (4) the total number of eggs produced (Poisson distribution, log link) by singly and multiply mated females of both species and the associated oviposition periods (Poisson distribution, log link), and (5) the sex ratio (arcsin square-root transformed before analysis; normal distribution, identity link) and number (binomial distribution, logit link) of offspring surviving to adulthood of singly and multiply mated females of both species.

#### Paternity analyses

Statistical analyses were carried out using SPSS 21 (IBM Corp., USA). The number of male mates siring at least one offspring of a given female (Gamma distribution, log link) was compared between *P*. *persimilis* and *N*. *californicus* by GLM. Before analysis, this number was calculated for each female by multiplying the observed number of matings with the mean proportional estimate of successful male mates, i.e. those siring at least one offspring, derived from genotyping all female offspring of 11 and 10 multiply mated females of *P*. *persimilis* and *N*. *californicus*, respectively. To analyze paternity success for the complete dataset of the mating experiment, including both singly and multiply mated females, we first generated an index of paternity success of first successful and later male mates for each female. The index of paternity success represents an estimate of the number of daughters (in these mites, only daughters carry both the maternal and the paternal chromosome set) sired by the first successful and later male mates of a given female, taking into account species-specific female re-mating likelihoods, offspring sex ratio and proportional share in siring offspring of first successful and later male mates. We used generalized estimating equations (GEE; normal distribution, identity link; autocorrelation structure between successful first and later males) to analyze the influence of species and male mate (successful first or later) on the index of paternity success.

## Results

### Mating and reproductive traits

**Fig 1:**
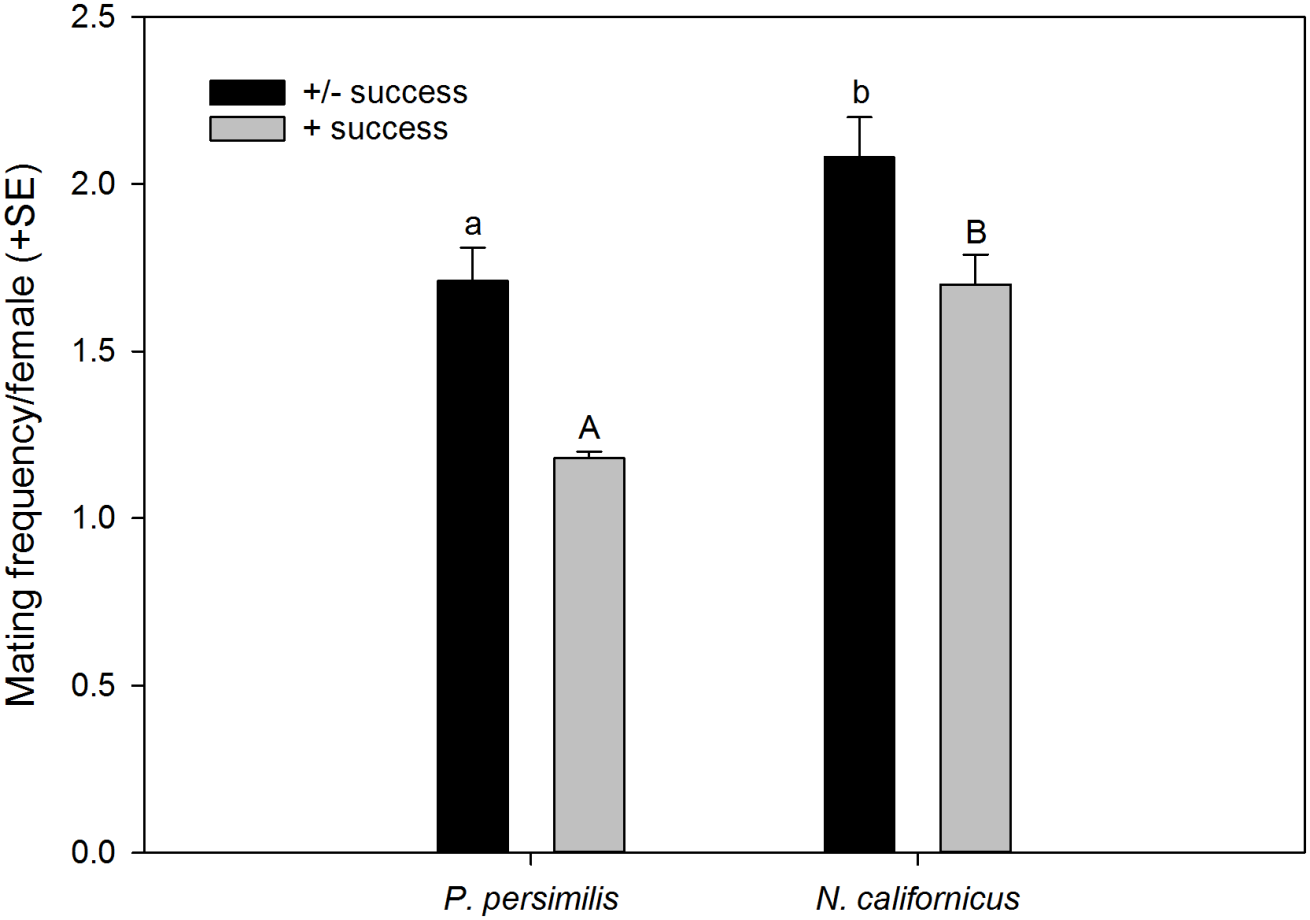
Mating frequency. Number of matings of females of *P*. *persimilis* (N = 30) and *N*. *californicus* (N = 23), including all male mates (independent of their paternity success; +/- success) and including only those male mates with paternity success (i.e. those siring at least one offspring of a given female; + success). Different letters on top of bars indicate significant differences between species within +/- success and + success (GLMs; *P* < 0.05).

**Fig 2:**
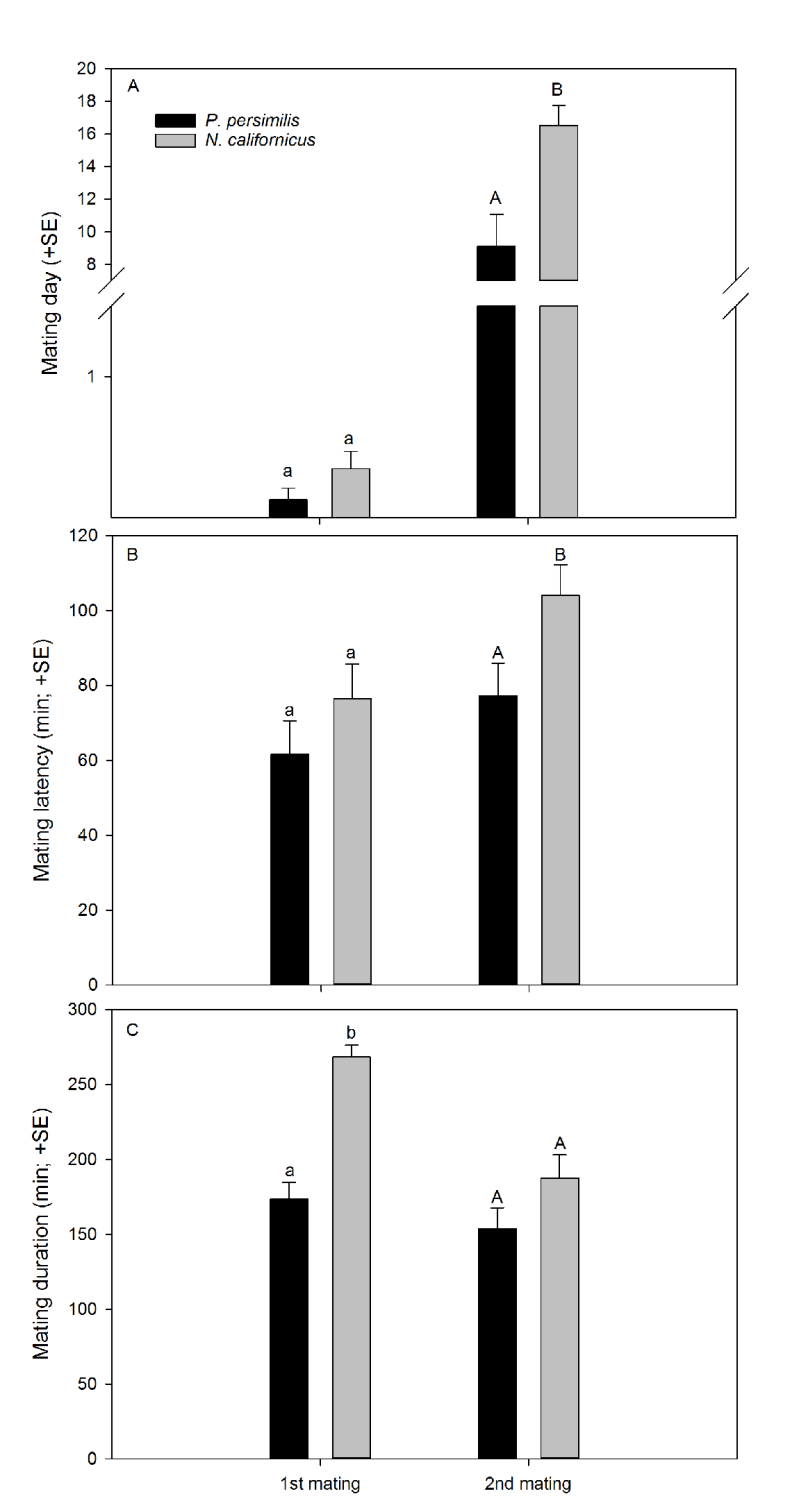
Mating dates (A), latency (B) and duration (C). (A) Dates, i.e. days after reaching adulthood, when females of *P*. *persimilis* and *N*. *californicus* accepted and successfully mated with their first and second mates; (B) mating latency, i.e. time elapsed until mating occurred after offering a male, of females of *P*. *persimilis* and *N*. *californicus* offered the first and second mates; (C) mating duration of females of *P*. *persimilis* and *N*. *californicus* with first and second mates. Male mates were offered on days 0 to 2, 9 to 13 and 17 to 23; N = 30 for *P*. *persimilis* and N = 23 for *N*. *californicus*. Different letters on top of bars indicate significant differences between species within the first and second mating (GLMs; *P* < 0.05).

**Fig 3:**
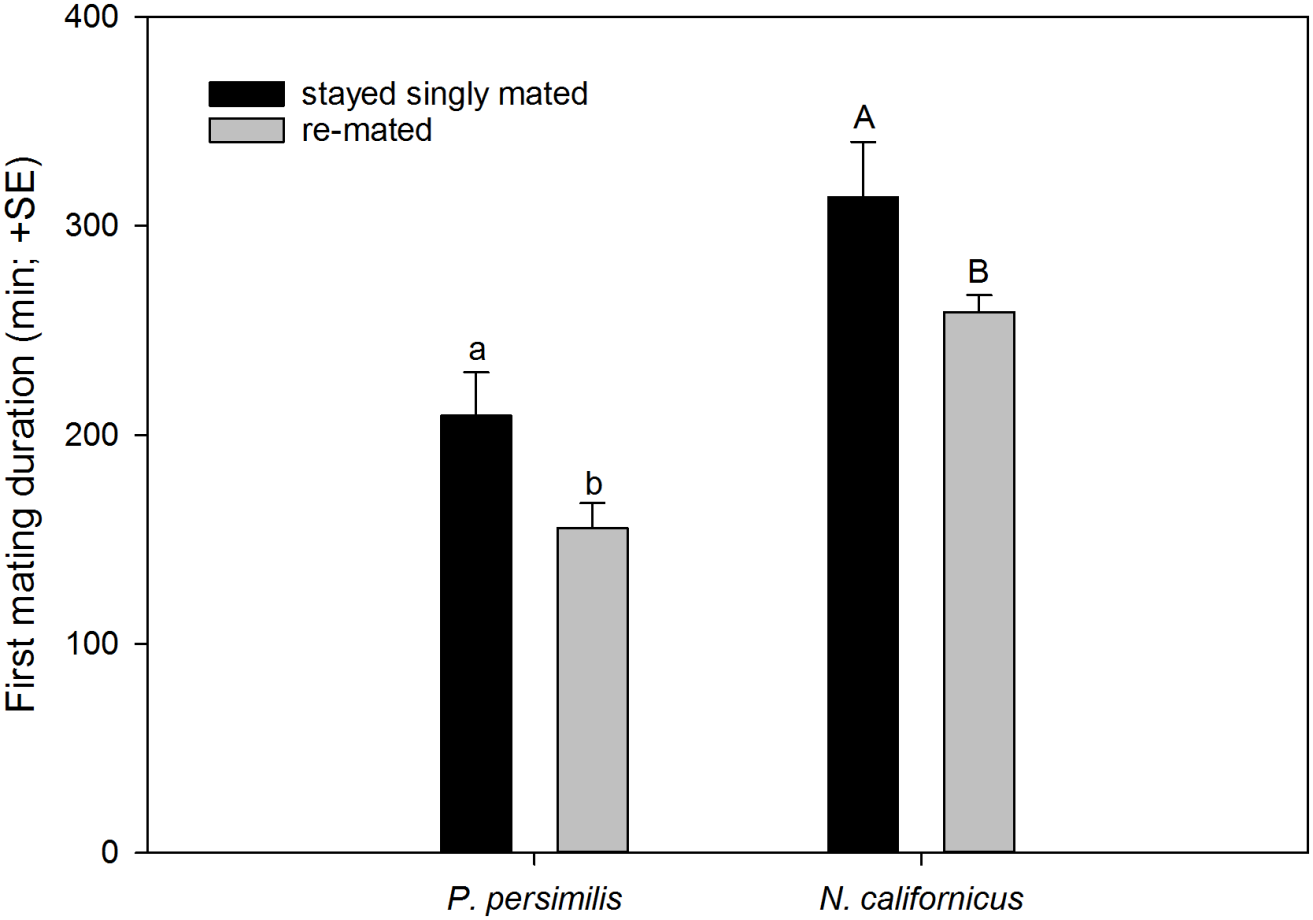
Re-mating propensity depends on duration of first mating. First mating duration of females of *P*. *persimilis* (N = 30) and *N*. *californicus* (N = 23), staying singly mated and re-mating, respectively. Different letters on top of bars indicate significant differences between singly mating and re-mating females within species (GLM; *P* < 0.05).

*Neoseiulus californicus* females re-mated more often than *P*. *persimilis* females (GLM; *Wald ӽ*^*2*^ = 5.875, *P* = 0.015; Fig 1), which was consistent among experimental series (*Wald ӽ*^*2*^ = 8.329, *P* = 0.215). *Phytoseiulus persimilis* and *N*. *californicus* females did not differ in the timing of first mate acceptance and mating (GLM; *Wald ӽ*^*2*^ = 2.023, *P* = 0.155) but re-mating, i.e. second mate acceptance and mating, occurred earlier in *P*. *persimilis* than *N*. *californicus* (GLM; *Wald ӽ*^*2*^ = 7.229, *P* = 0.007) (Fig 2A). First mating latency was similar in both species (GLM; *Wald ӽ*^*2*^ = 1.038, *P* = 0.308; Fig 2B) and independent of females later re-mating or not (GLM; *Wald ӽ*^*2*^ = 0.474, *P* = 0.491) and of the interaction between species and re-mating or not (*Wald ӽ*^*2*^ = 0.113, *P* = 0.736). In contrast, second mating latency was longer in *N*. *californicus* than *P*. *persimilis* (GLM; *Wald ӽ*^*2*^ = 5.294, *P* = 0.021) (Fig 2B). *Neoseiulus californicus* females mated longer with their first mate than *P*. *persimilis* females (GLM; *Wald ӽ*^*2*^ = 43.857, *P* < 0.001). Second mating duration was marginally significantly longer in *N*. *californicus* than *P*. *persimilis* (*Wald ӽ*^*2*^ = 2.854, *P* = 0.097) (Fig 2C). In both species, females that re-mated, mated shorter with their first mates than females that stayed singly mated (*Wald ӽ*^*2*^ = 11.970, *P* < 0.001) (Fig 3). This pattern was similarly expressed in both species, as indicated by the non-significant interaction between species and re-mating or not (*Wald ӽ*^*2*^ = 0.001, *P* = 0.969).

**Fig 4:**
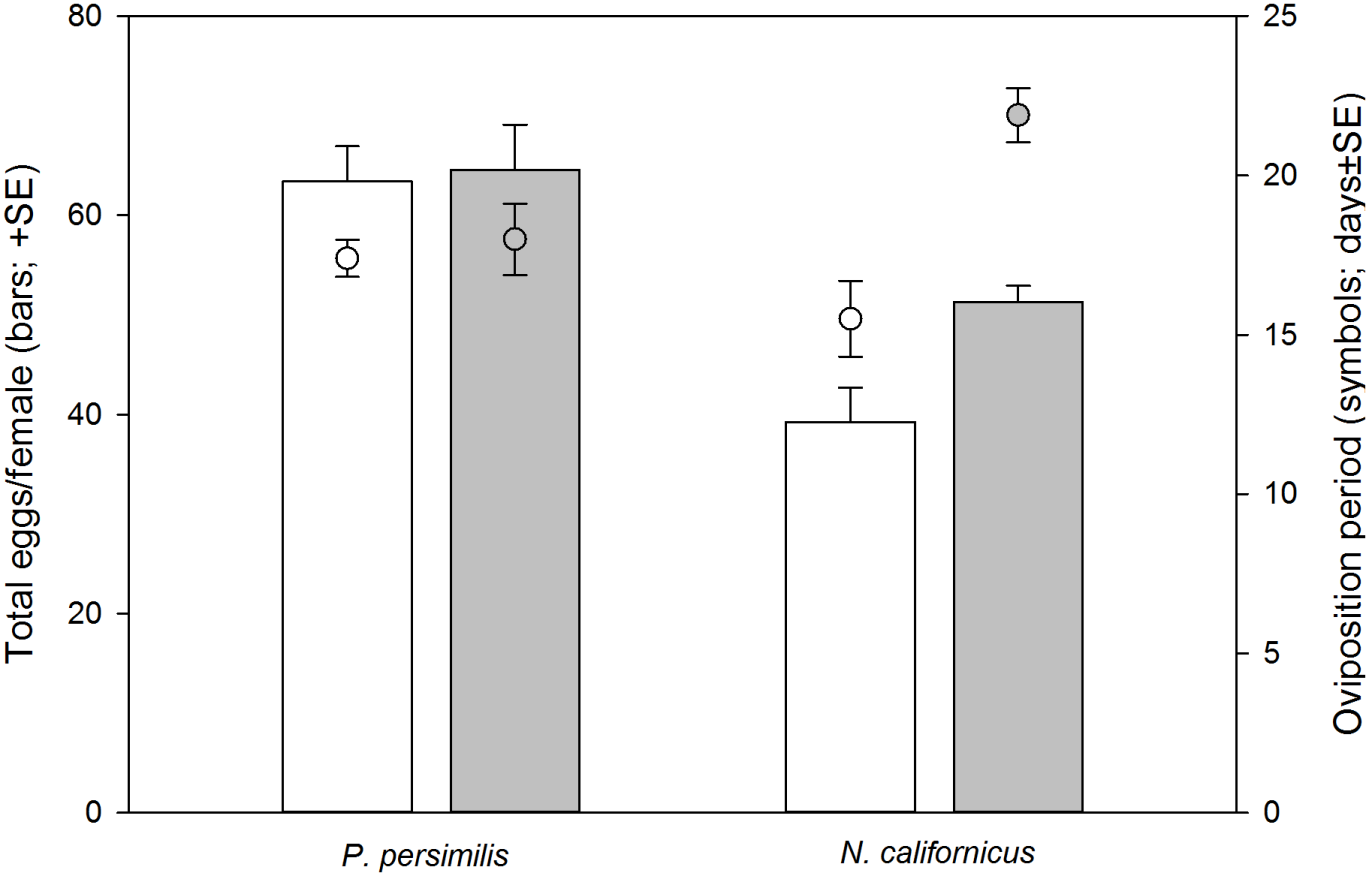
Oviposition. Total number of eggs produced (bars) and oviposition period (symbols) of singly and multiply mated females of *P*. *persimilis* (N = 30) and *N*. *californicus* (N = 23). GLM for total number of eggs produced: species (*Wald ӽ*^*2*^ = 51.710, *P* < 0.001), single/multiple mating (*Wald ӽ*^*2*^ = 8.332, *P* = 0.004), species*single/multiple mating (*Wald ӽ*^*2*^ = 6.363, *P* = 0.012). GLM for oviposition days: species (*Wald ӽ*^*2*^ = 0.610, *P* = 0.435), single/multiple mating (*Wald ӽ*^*2*^ = 13.622, *P* < 0.001), species*single/multiple mating (*Wald ӽ*^*2*^ = 9.187, *P* = 0.002).

*Neoseiulus californicus* and *P*. *persimilis* had similarly long oviposition periods (GLM; *Wald ӽ*^*2*^ = 0.610, *P* = 0.435). The oviposition period of multiply mated females was longer than that of singly mated females (*Wald ӽ*^*2*^ = 13.622, *P* < 0.001) in *N*. *californicus* but not in *P*. *persimilis* (species*single/multiple mating; *Wald ӽ*^*2*^ = 9.187, *P* = 0.002) (Fig 4). *P*. *persimilis* produced more eggs than *N*. *californicus* (*Wald ӽ*^*2*^ = 51.710, *P* < 0.001). Multiply mated females produced more eggs than singly mated females (*Wald ӽ*^*2*^ = 8.332, *P* = 0.004) in *N*. *californicus* but not in *P*. *persimilis* (species*single/multiple mating; *Wald ӽ*^*2*^ = 6.363, *P* = 0.012) (Fig 4). Offspring sex ratio was more biased towards females in *P*. *persimilis* (female proportion mean ± SE; 0.84 ± 0.03) than *N*. *californicus* (0.74 ± 0.01) (*Wald ӽ*^*2*^ = 4.207, *P* = 0.040) but was unaffected by single/multiple mating (*Wald ӽ*^*2*^ = 1.795, *P* = 0.180) and its interaction with species (*Wald ӽ*^*2*^ = 0.283, *P* = 0.595). The proportion of offspring surviving to adulthood was higher in *P*. *persimilis* than *N*. *californicus* (*Wald ӽ*^*2*^ = 6.197, *P* = 0.013) and not influenced by single/multiple mating as main factor (*Wald ӽ*^*2*^ = 2.231, *P* = 0.135). However, the significant interaction (*Wald ӽ*^*2*^ = 5.067, *P* = 0.024) indicates that in *N*. *californicus* offspring of multiply mated females (mean ± SE; 0.79 ± 0.02) had better survival chances than those of singly mated females (0.72 ± 0.09), whereas in *P*. *persimilis* the survival chances of offspring of singly (0.81 ± 0.04) and multiply (0.81± 0.02) mated females were similar.

### Paternity analyses

**Fig 5:**
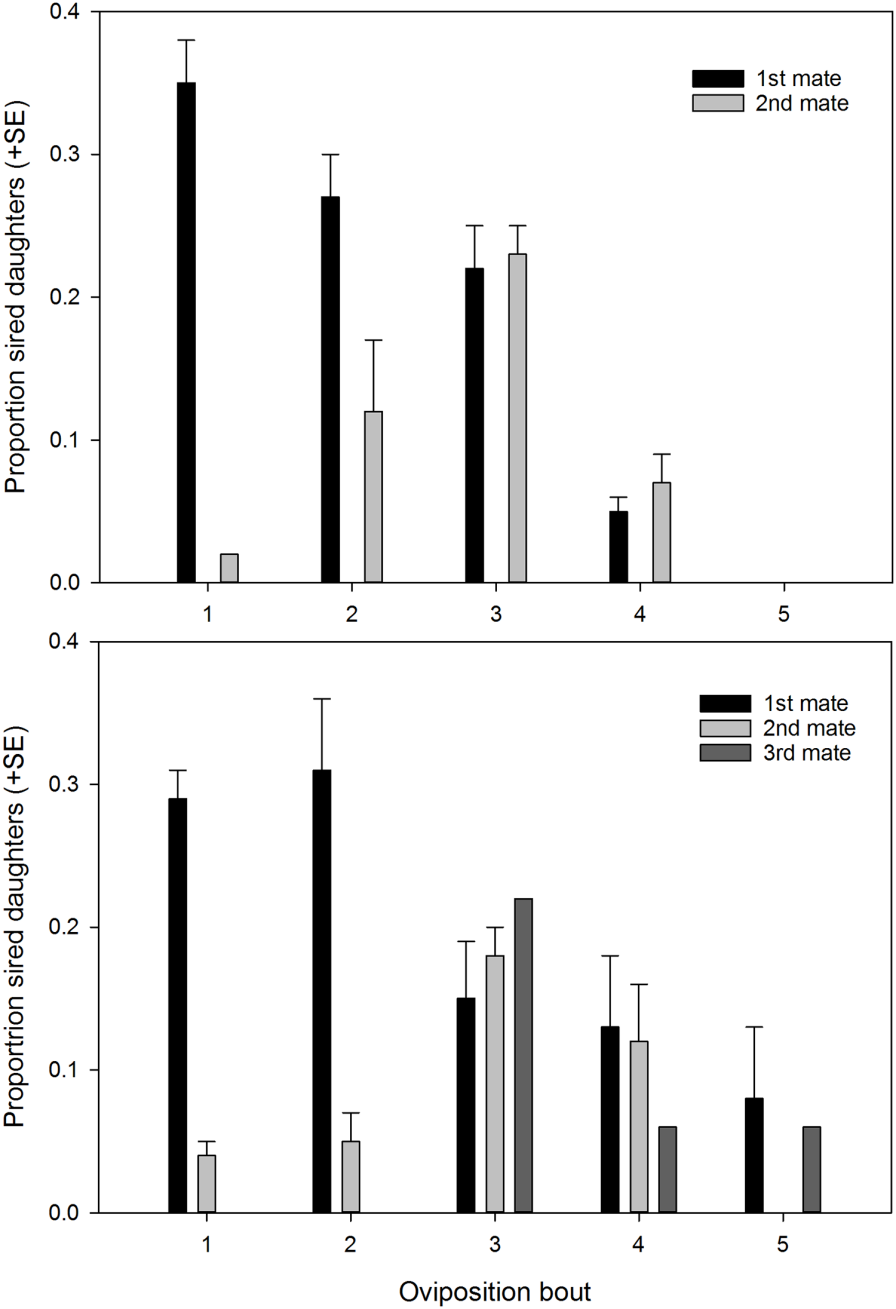
Proportional paternity of male mates. Proportion of daughters from multiply mated females of *P*. *persimilis* (N = 20) and *N*. *californicus* (N = 19) sired by first, second and third male mates over time. Egg collection started on the first day after mating; for rearing offspring to adulthood, eggs laid over 6 day periods (oviposition bouts) were placed on the same leaf arena.

Considering the dataset subjected to genotyping, 5 of 11 and 7 of 10 multiply mated females of *P*. *persimilis* and *N*. *californicus*, respectively, were truly polyandrous, i.e. offspring were sired by at least two males. Two females of *N*. *californicus* produced offspring sired by three male mates each, whereas *P*. *persimilis* females had at maximum two males siring offspring (Fig 5). In three *P*. *persimilis* females, first male mates did not sire any offspring, which was not observed in *N*. *californicus*. First successful male mates sired 87.5 ± 0.063 (%; mean ± SE) and 80 ± 0.068 offspring in *P*. *persimilis* and *N*. *californicus*, respectively.

**Fig 6:**
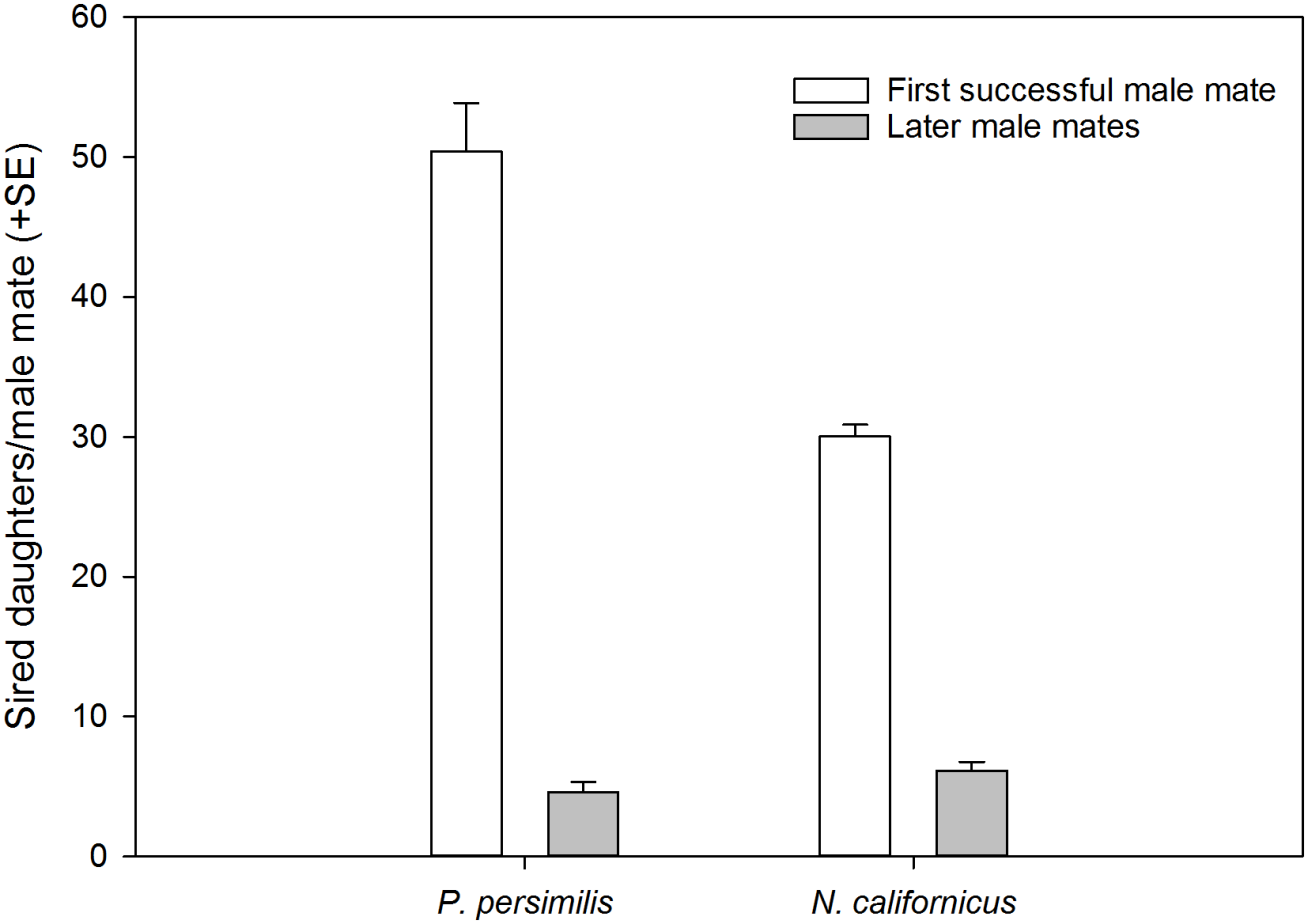
Paternity success index. Estimated number of sired daughters of first successful and later male mates of *P*. *persimilis* (N = 20) and *N*. *californicus* (N = 19). Results of GEE: species *(Wald ӽ*^*2*^ = 17.906, *P* < 0.001), male mate (Wald ӽ^2^ = 330.195, *P* < 0.001), species*male mate *(Wald ӽ*^*2*^ = 32.741, *P* < 0.001).

Considering both singly and multiply mated females of the whole dataset of the mating experiment, the number of male mates siring at least one offspring per female (Fig 1; + success) was higher in *N*. *californicus* than *P*. *persimilis* (GLM; *Wald ӽ*^*2*^ = 21.146, *P* < 0.001). The paternity success index (estimated number of sired daughters) was higher in *P*. *persimilis* than *N*. *calfornicus* males (GEE; *Wald ӽ*^*2*^ = 17.906, *P* < 0.001) and in both species higher in first successful than later male mates (Wald ӽ^2^ = 330.195, *P* < 0.001). However, the significant interaction (*Wald ӽ*^*2*^ = 32.741, *P* < 0.001) indicates that first successful males sired more daughters in *P*. *persimilis* than *N*. *californicus* whereas later males sired more daughters in *N*. *californicus* than *P*. *persimilis* (Fig 6).

## Discussion

The adaptive significance of polyandry corresponded well to the level of polyandry in the predatory mites *N*. *californicus* and *P*. *persimilis*. Ultimately, polyandry yielded clear direct and indirect fitness benefits in *N*. *californicus*: multiply mated females produced more offspring over longer times with higher survival chances than singly mated females, which is similar to the findings for *Drosophila pseudoobscura* [29]. The only obvious fitness benefit in *P*. *persimilis* was fertilization assurance in females where the first mating partners completely failed in fertilizing offspring. Possible reasons for such failures include behavioral deficiency in mating and sperm transfer, despite the mating durations being well within the range of time needed for successful and complete insemination [21, 23, 26], and/or deficiency in sperm quality. Transferred sperm mass and number of spermatophores are unaffected by the mating duration above the minimum duration threshold (Walzer & Schausberger unpublished data). Multiple mating resulted in mixed offspring paternities in both species opening the chance for genetic benefits. However, paternity success of later male mates and genotypic offspring variability were higher in *N*. *californicus* than *P*. *persimilis*. Possible indirect genetic fitness benefits [9, 10], applicable to both species, are enhanced viability, genetic compatibility, genetic variability or genetic complementarity [30], but need yet to be scrutinized. Mixed paternity could for example help to mitigate the costs of sib-mating and inbreeding among offspring [31], or could enhance the offspring survival chances in unpredictable environments, as bet-hedging strategy. Inbreeding is a more serious threat in the group-living *P*. *persimilis* [32], which is highly specialized to exploit patchily distributed spider mites as prey, than in the more dispersedly living generalist predator *N*. *californicus*. Provided sufficient prey supply, *P*. *persimilis* young tend to stay in their natal patch until adulthood [27, 33]. In such a scenario, mixed paternity together with well-developed social recognition abilities [25, 34, 35] are proper strategies to reduce the risk of inbreeding depression.

Regarding possible proximate factors, a comparably short duration of the first mating, albeit above the threshold needed for complete successful insemination, which is, at 25°C, about 80 min in *P*. *persimilis* and 150 min in *N*. *californicus* [21, 26], apparently increased the female re-mating propensity in both species. Mating duration may indicate male mate quality or compatibility. In arthropods, including mites, high sexual activity of females commonly entails considerable time- and energy-related costs such as shortening female longevity [24, 36, 37], unless the males provide nutrients through mating and thereby increase female longevity [38, 39]. Thus, to optimally balance the benefit/cost tradeoff of re-mating, females of *P*. *persimilis* probably have a lower re-mating propensity than those of *N*. *californicus* because of poorer (no increase in egg numbers) and less predictable (context-dependency of indirect benefits) re-mating benefits. Females of *N*. *californicus* re-mated on average later than females of *P*. *persimilis*, indicating that, additionally to the first mating duration, the females’ re-mating willingness was probably internally stimulated by sperm depletion, aging, or inactivity. This explanation is also supported by the findings that re-mating may revive oviposition in older *N*. *californicus* females, which only seemingly ceased egg production [26]. Similar re-mating and reproductive patterns were observed in the closely related *N*. (*Amblyseius*) *womersleyi* [40]. Early re-mating by *P*. *persimilis* females, which was only observed in a few *N*. *californicus* females, suggests that those females more strongly targeted indirect genetic benefits rather than increasing numbers or other immediate direct benefits. Shorter mating latency in the low-level polyandrous *P*. *persimilis* than the medium-level polyandrous *N*. *californicus* corresponds to the general trend of a positive correlation between the timing and frequency of mating in insects [41]. Considering that age at first reproduction is a key life history trait commonly traded off against longevity and *N*. *californicus* lives longer than *P*. *persimilis*, early mating likely has higher fitness relevance in *P*. *persimilis* than *N*. *californicus*.

Due to the lower level of polyandry, the operational sex ratio (ratio between the number of receptive females and sexually active males) is more strongly skewed towards males in *P*. *persimilis* than *N*. *californicus* [23]. Thus, males of *P*. *persimilis* are under stronger selective pressure for pre-mating competitive ability than those of *N*. *californicus*. Our study revealed that the relative paternity success of later male mates is higher in *N*. *californicus* than that in *P*. *persimilis* (later male mates sired a higher proportion of daughters), relaxing the pressure to out-compete rival males before mating and to be the first mate accepted by a female, respectively. Male competition is also influenced by male body size [21], but much more so in *P*. *persimilis* than *N*. *californicus*, ultimately explaining differing male body size plasticity [23, 27]. While higher levels of polyandry relaxes selection pressure on competitive ability before mating, because each male has more chances to mate, it increases selection pressure on post-copulatory mechanisms [42], assuring paternity in multiply mated females, such as sperm competition [12] or guarding/defending the mated female [43]. If females mate only once then here is no need to be good in post-copulatory competition but if they re-mate, mechanisms such as sperm competitive ability count. The intensity of post-copulatory male-male competition has not yet been compared between *P*. *persimilis* and *N*. *californicus* but should be higher in the latter.

## Conclusions

Our study provides a key example for linking behavioral experiments, quantification of reproductive traits and genotyping to explain the evolution of differing levels of polyandry and is the first to link polyandry and parentage analyses in phytoseiid predatory mites. Thus far, it was only known that re-mating increases offspring number in species such as *N*. *californicus* [26], *N*. *womersleyi* [40], *N*. *cucumeris* [44], *Kampimodromus aberrans* [45] or *Amblyseius andersoni* [24] but it was unclear whether this is due to stimulated or enhanced egg production by the mating act or accessory seminal fluids or indeed due to additional paternity by later male mates. Our study provides rigorous evidence for the latter explanation in *N*. *californicus*. Identification of polymorphic microsatellite loci, occurrence of graded levels of polyandry, and great experimental suitability make phytoseiid predatory mites easily accessible model organisms for studying the evolution of female mating systems, analogous to the currently most comprehensively studied animals in this regard, *Drosophila* sp. [7]. In both predatory mite species we observed three basic types of females, monandrous, pseudo-polyandrous and truly polyandrous. To which extent in *P*. *persimilis* within-population between-individual behavioral flexibility in re-mating [16] represents phenotypic plasticity, conditionally depending on proximate aspects such as male size [21], compatibility in genetic relatedness [25], or risk of inbreeding within local groups, or genotypically fixed mixed strategies, or a combination of both, requires further scrutiny.

## Supporting Information

S1 File Raw data of the mating experiment. (XLS)

S1 Table Genotypes used for paternity analysis in *P*. *persimilis*. (DOCX)

S2 Table Genotypes used for paternity analysis in *N*. *californicus*. (DOCX)
