# Supplementary material for: Ultimate Drivers and Proximate Correlates of Polyandry in Predatory Mites
Source: PLoS One. 2016 Apr 21;11(4):e0154355. doi: 10.1371/journal.pone.0154355 (PMC4839743; doi:10.1371/journal.pone.0154355)
Supplement: S1 Table — (DOCX) [file pone.0154355.s002.docx]

**Supplementary table 1**

Ultimate drivers and proximate correlates of polyandry in predatory mites

Peter Schausberger, J. David Patiño-Ruiz, Masahiro Osakabe, Yasumasa Murata, Naoya Sugimoto, Ryuji Uesugi, Andreas Walzer

**Supplementary table 1**. Genotypes of females and 1^st^, 2^nd^, and 3^rd^ male mates used for paternity analysis of *P. persimilis.*

| Female ID | Sex and mate order | Loci and alleles^1^ | |
| --- | --- | --- | --- |
|  |  | PP003 | PP005 |
| PP 1 | Female | 124/124 | 272/272 |
|  | 1^st^ male | 124* | 272 |
|  | 2^nd^ male | 122* | 266 |
| PP 5 | Female | 124/124 | 272/272 |
|  | 1^st^ male | no peak | 266* |
|  | 2^nd^ male | 124 | 272* |
| PP 6 | Female | 124/124 | 266/266 |
|  | 1^st^ male | 124* | 266 |
|  | 2^nd^ male | 122* | 266 |
| PP 9 | Female | 124/124 | 266/266 |
|  | 1^st^ male | 124 | 272* |
|  | 2^nd^ male | 122 | 266* |
| PP11 | Female | 122/122 | 266/266 |
|  | 1^st^ male | 124* | 266 |
|  | 2^nd^ male | 122* | 266 |
| PP16 | Female | 124/124 | 266/266 |
|  | 1^st^ male | 122 | 266* |
|  | 2^nd^ male | 124 | 272* |
| PP17 | Female | 124/124 | 266/266 |
|  | 1^st^ male | 122* | 266 |
|  | 2^nd^ male | 124* | 266 |
| PP22 | Female | 124/124 | 272/272 |
|  | 1^st^ male | 122* | 266 |
|  | 2^nd^ male | 124* | 266 |
| PP26 | Female | 124/124 | 272/272 |
|  | 1^st^ male | 124* | 266* |
|  | 2^nd^ male | 124 | 272* |
|  | 3^rd^ male | 122* | 266* |
| PP28 | Female | 124/124 | 272/272 |
|  | 1^st^ male | 122* | 266 |
|  | 2^nd^ male | 124* | no peak |
| PP34 | Female | 122/122 | 266/266* |
|  | 1^st^ male | 124 | 266* |
|  | 2^nd^ male | 124 | 272* |

^1^Asterisks represent diagnostic alleles used for paternity determination.
