# Supplementary material for: Ultimate Drivers and Proximate Correlates of Polyandry in Predatory Mites
Source: PLoS One. 2016 Apr 21;11(4):e0154355. doi: 10.1371/journal.pone.0154355 (PMC4839743; doi:10.1371/journal.pone.0154355)
Supplement: S2 Table — (DOCX) [file pone.0154355.s003.docx]

**Supplementary table 2**

Ultimate drivers and proximate correlates of polyandry in predatory mites

Peter Schausberger, J. David Patiño-Ruiz, Masahiro Osakabe, Yasumasa Murata, Naoya Sugimoto, Ryuji Uesugi, Andreas Walzer

**Supplementary table 2**. Genotypes of females and 1^st^, 2^nd^, and 3^rd^ male mates used for paternity analysis of *N. californicus.*

| Female ID | Sex and mate order | Loci and alleles^1^ | |
| --- | --- | --- | --- |
|  |  | NC19 | NC030 |
| NC4 | Female | – | 235/235 |
|  | 1^st^ male | – | 237* |
|  | 2^nd^ male | – | 235* |
| NC11 | Female | 164/182 | 237/237 |
|  | 1^st^ male | 182 | 235* |
|  | 2^nd^ male | 182 | 237* |
| NC13 | Female | – | 237/237 |
|  | 1^st^ male | – | 235* |
|  | 2^nd^ male | – | 237* |
| NC21 | Female | 162/182 | 235/235 |
|  | 1^st^ male | 182 | 235* |
|  | 2^nd^ male | 164* | 237* |
|  | 3^rd^ male | 182* | 237* |
| NC22 | Female | 162/182 | 235/235 |
|  | 1^st^ male | 178* | 235 |
|  | 2^nd^ male | 182* | 237* |
|  | 3^rd^ male | 162* | 235* |
| NC24 | Female | – | 229/237 |
|  | 1^st^ male | – | 235* |
|  | 2^nd^ male | – | 237* |
| NC25 | Female | – | 229/229 |
|  | 1^st^ male | – | 235* |
|  | 2^nd^ male | – | 237* |
| NC26 | Female | – | 229/229 |
|  | 1^st^ male | – | 237 |
|  | 2^nd^ male | – | 235 |
| NC30 | Female | – | 235/235 |
|  | 1^st^ male | – | 235* |
|  | 2^nd^ male | – | 237* |
| NC37 | Female | 182/182 | 229/235 |
|  | 1^st^ male | 182 | 237* |
|  | 2^nd^ male | 182* | 235* |
|  | 3^rd^ male | 162* | 229* |

^1^Asterisks represent diagnostic alleles used for paternity determination.
